# Effectiveness of mobile apps to improve urinary incontinence: a systematic review of randomised controlled trials

**DOI:** 10.1186/s12912-022-00812-6

**Published:** 2022-01-28

**Authors:** Renee Widdison, Amineh Rashidi, Lisa Whitehead

**Affiliations:** School of Nursing and Midwifery, Edith Cowan University, 270 Joondalup Drive, Joondalup, WA 6027 Australia

**Keywords:** M-health, Mobile applications, Mobile apps, Pelvic floor muscle training, Adherence, Urological

## Abstract

**Introduction:**

Pelvic floor exercises are effective in the treatment of urinary incontinence (UI) and are routinely prescribed, along with bladder training, by primary healthcare providers as first line conservative management. Mobile phone applications are increasingly popular within the healthcare setting and can provide opportunities for patients to complete treatments at home. To date, there has not been a systematic review examining outcomes from randomised controlled trials on the effectiveness of mobile applications to improve UI.

**Methods:**

A systematic review of randomized controlled trials evaluating the effectiveness of mobile applications to improve UI was carried out according to the PRISMA reporting guidelines. The online databases MEDLINE, Embase, PsychINFO, CINAHL, Web of Science, Scopus, The Cochrane Library, Joanna Briggs Institute (JBI), Google Scholar were searched for papers published between 2007 to 2020. Keywords and MeSH terms were used to identify relevant English language studies. The quality and risk of bias within included studies was assessed by two independent reviewers, RCT JBI critical appraisal tool. Due to heterogeneity in the outcome of studies, a meta-analysis of the data could not be conducted.

**Findings:**

Four studies reported an improvement in the outcome assessed post-intervention, suggesting that using mobile phone applications for pelvic floor muscle training (PFMT) was an acceptable and valid intervention to improve UI.

**Conclusion:**

Mobile applications for PFMT indicated that increase adherence to treatment and decrease UI. The integration of this treatment modality into current practice is recommended. Mobile phone applications for PFMT show promise in the conservative management of UI. Further research is required to support the use of this technology in the conservative management of UI.

**Supplementary Information:**

The online version contains supplementary material available at 10.1186/s12912-022-00812-6.

## Background

An estimated 400 million adults worldwide experience urinary incontinence (UI) [1]. Pelvic floor muscle training (PFMT) is a conservative first line treatment for UI defined as the involuntary loss of urine [2]. There is evidence emerging from randomized controlled trials signifying improvement of UI and quality of life (QOL) by adherence to pelvic floor training. Pelvic floor muscles are like a funnel and contain the Levator ani muscles and further divided into the Puborectalis, Pubococcygeus, Iliococcygeus, alongside the Coccygeus muscle and supporting fascia. These muscles support the visceral organs including the bladder, urethra, uterus, and bowel and are responsible for maintaining continence [3]. Pelvic floor muscle weakness is as a disorder affecting both men and women which contributes to UI. Conditions contributing to weak pelvic floor muscles and UI include obesity, constipation, pregnancy, childbirth, menopause, heavy lifting, surgery, trauma, and radiation treatment [4]. Strengthening the pelvic floor muscles can be achieved through a set of exercises specifically targeting muscle groups.

Currently, pelvic floor strength and contraction duration remain important measurements in pelvic floor assessment. PFMT exercises offer conservative management for UI and are routinely prescribed for treatment of UI, in conjunction with improved fluid intake management and bladder habits. Patients are required to complete exercises daily at home as part of their treatment plan. Recommended guidelines with high evidence of effectiveness exist in current practice [5] Health care professionals have access to these guidelines however they do not always follow them, and locally developed programs exist instead [5]. The traditional method of face to face training disadvantages some people from participation in training programs. Uptake to training programs is notably affected by service provision and challenges accessing services [6]. PFMT delivered via mobile applications are easily accessed by people in the home setting and have been found to be advantageous in overcoming known barriers to training whilst also offering the same outcomes as stand-alone practice [7]. Recent studies indicate that mobile applications for the treatment of UI can be of value in future treatment [7]. Mobile applications have been reported as user friendly, with the capability of saving favourite exercise routines and can act as reminder for personalised exercise [7]. The functions within the mobile application enabled and encouraged participants, who have saved the correct number of repetitions and obtained optimal results and satisfaction [7]. There is yet to be a systematic review of the effectiveness of mobile application used to improve UI. Therefore, this review extends the current literature providing a narrative synthesis of the most recent randomised controlled trials evaluating mobile applications to deliver PFMT for UI.

### Aim

This review aimed to synthesize evidence from randomised controlled trials to examine the effectiveness of mobile applications delivering PFMT to improve UI.

## Methods

### Search strategy and inclusion criteria

The searches were conducted in MEDLINE, Embase, PsychINFO, CINAHL, Web of Science, Scopus, The Cochrane Library, Joanna Briggs Institute (JBI), and Google Scholar. The search was performed using the following key search terms: pelvic floor exercise*, mobile app*, Kegel exercise*, mobile application*(Appendix 1). A priori eligibility criteria identified in the protocol was used to identify studies for inclusion**.** The following eligibility criteria was considered in this review: (1) Population: identify as adults over 18 years and diagnosis of urinary incontinence; (2) Intervention: PFMT using mobile phone applications (3) Outcomes: Primary Outcome; adherence to pelvic floor muscle training. Secondary Outcomes; Perceived value, Quality of Life (QoL) and symptom improvement. (4) Study Design: Randomised controlled trial (RCT). Studies in the English language, peer-reviewed studies between 2007 and 2020 were included. Studies with paediatric or adolescent UI, and prior to 2007 were also excluded as mobile phone applications were not available. Initially, (*n* = 416) articles were identified and *n* = 270 articles were removed as duplicates (Appendix 2). First and second reviewers performed title and abstract screening, then they conducted full text screening [8].

### Quality appraisal and data extraction

Risk of bias and methodological quality were assessed for each study using the JBI Critical Appraisal Checklist for Randomised Controlled trials, which contained 13 items [8]. Two reviewers independently appraised the risk of bias and methodological quality for each included study as yes, no, unclear, or not applicable. Data were extracted, using the JBI standardised data extraction tools by RCT study design. Specific details about the sample size, sample demographics (age and sex), intervention details, setting, location, data collection, data analysis study design, data source or measurement of outcomes of interest, and key findings were extracted.

### Data synthesis

Key findings were narratively synthesised to demonstrate the impact of PFMT delivered via a mobile application on the primary and secondary outcomes. Meta-analysis was not completed due to heterogeneity in data collection time points, source of data and study outcomes. Therefore, a narrative synthesis was conducted according to the Synthesis Without Meta-analysis (SWiM) guidelines [9].

## Results

### Study inclusion

The published studies were between 2016 and 2020 and were undertaken in Sweden, Canada, Brazil, and China. Of the four trials, three were complete trials, and one a two-year follow-up post completion of an RCT. A variety of primary and secondary outcomes were assessed across the trials. Outcomes documented in the trials were adherence to PFMT, symptom severity, QOL, bladder neck function, sexual activity, cure rates and use of incontinence products. Three studies [10–12] included symptom severity and adherence as primary outcomes. One study [13] continued with the same outcomes from the Asklund, Nyström [11] trial as they conducted the 2 year follow up. Each study clearly defined the primary and secondary outcomes and time to follow-up varied across the studies.

### Quality of included studies

The JBI quality appraisal tool, a 13-point checklist for randomised controlled trials was used to assess the quality of the four studies (Appendix 3). The methodological quality of the studies was assessed based on methods of randomisation, allocation concealment, blinding, outcomes, statistical analysis, and loss to follow-up. True randomisation, allocation concealment and similar baseline were unanimous across the studies except for the two-year follow-up trial. Blinding was true amongst all participants in the studies conducted by two studies [10, 11]. Blinding for participants was declared not feasible by two studies [12, 13]. Those delivering treatments were blinded in only one study [12]. True blinding of outcome assessors did not occur in the four studies.

### Review findings

The findings synthesised into three categories, appendix 4 presents an overview of the study characteristics.

#### Adherence to PFMT using a mobile application and perceived value of using a mobile application

Data from four studies contributed to this finding [10–13]. The four studies determined the success of adherence to PFMT using a mobile application by evaluating self-reported measures, urinary symptoms, app utilisation and self-efficacy. The study by Araujo et al. (2020) reported higher adherence rates in the mobile application group 1-2 months post the onset of the intervention (*P* < 0.001). One study reported a higher adherence rate in the mobile application group, they report that 41% performed pelvic floor exercises daily and this was vastly different to the control group who recorded a 3.3% adherence rate to daily pelvic floor exercise [11]. A study by Hoffman and colleagues [13] followed up the original study [11] and of the 46 women who participated in the follow-up, all 46 women had downloaded and kept the mobile application and 17.4% were still using it 2 years later. Frequent users who intend to use mobile applications on a regular basis, indicated higher PFMT adherence compared to those performing stand-alone exercises … *“based on our results women who used it not only exercised more than the other group, based on exercise sections duration but also felt more committed to exercising”*(10, p.6). Also, people who used mobile applications in the short term were satisfied about the outcome: *“with the present study we have shown the app treatment to be effective in the short term*” (11, p.1375). The high-level adherence rate of PFMT using an application resulted in participants suffering less from pelvic floor injury, less bladder neck descent and improved pelvic floor strength: *“participants receiving the app-based audio guidance indicated more positive effect to 6 months postpartum”* (12,p.7). Patients who used mobile applications to undertake exercise reported positive aspects of the application: *“the reminder notes were important to exercise adherence and preferred to continue using the said feature”* (10, p.6).

#### QOL

Findings from three studies, [10, 11, 13] contributed to this synthesised finding. QOL measures are an estimation of wellbeing which can be assessed by measuring improvements in areas like healthcare and are based on an individual’s perception and expectations of treatment outcomes. For example, a study by Asklund [11], considered condition-specific QOL. In this study, the majority of participants in the intervention group (98.4%) were still engaged in the PFMT at follow-up, and 41.0% (25/61) performed PFMT daily. In comparison to the control group, 26.7% reported that they had not performed any PFMT and only 3.3% had performed PFMT daily. Therefore, self-reported QOL was higher in the intervention group compared to the control group, the groups significantly different (*P* < 0.001) [11]. Improvement in QOL 2 years post intervention was reported in another study, but there was no between the application group and control groups (*P* = 0.003) [10]. It is also reported that application group maintained the results of PMFT (*P* < 0.001) for a longer time which reflected on the improvements of their QOL [10]. The use of incontinence protection products reduced and almost 67% of participants reported that their leakage improved, and they used less incontinence protection products [13]. Discovering long term benefits of PFMT delivered via an app is an area for further study where to date the research has … “*yielded promising long-term results in terms of a condition that can severely affect QOL”* [13, p.1185].

#### Symptom improvement

Findings from three studies [10–12] contributed to the synthesised finding of symptom improvement. Symptom reduction measured by validated assessment tools and methods provides valuable data and true insight into the condition *“people experience greater improvement in symptom severity with a mean reduction in ICIQ scores”* (11,p. 1372) and *“we found that PFMT improves symptoms of UI, according to validated questionnaires”* (10,p.6). Furthermore, the cost and burden associated with continence aids is high and patients frequently ask during consultation when they will be able to cease using pads [11]. *“Outcome measurements at follow-up determined 56% of the app group as no leakage or>50% fewer leakage episodes than at baseline”* (11,p. 1372). Physically, a strong pelvic floor can improve and maintain continence as well as delay surgical intervention and the associated risks of major surgery [12]. Pelvic floor strength and less bladder neck descent has been noted in frequent users of mobile applications for pelvic floor muscle training: *“participants had significant improvements in symptom severity after 3 months”* (12, p.5), reiterating the practicality of an accessible PFMT program and the importance of conservative management of UI.

## Discussion

The purpose of this review was to synthesise the evidence on the effectiveness of mobile applications to improve adherence to PFMT. Four randomised controlled trials were included, and all four randomised controlled trials were considered high quality according to the JBI randomised controlled trial critical appraisal checklist [8]. The heterogeneity of the included studies resulted in a narrative synthesis. This review yielded 87 findings that led to the generation of four categories and three syntheses. Adherence to PFMT was key to effective treatment outcomes [10–13]. The success and effectiveness of treatment outcomes using a mobile application is attributed to adherence and a mobile application provides flexibility enabling the patient to take control and manage their condition at home. For example, a relatively new and attractive treatment modality provides reminder functions, feedback data, audio guidance and most importantly privacy. High level adherence had a direct impact on symptom reduction and improved QOL and these outcomes are of particular interest to health care providers aiming to provide results to their patients. Symptom reduction and improved QOL is a shared goal for both the provider and patient [14]. Such integration would require health promotion focussing on the established benefits and emphasising high level adherence to PFMT is achievable with a mobile application [15].

Mobile applications provide an alternative to regular pelvic floor muscle training, reduce symptoms and particularly provide opportunity to overcome health care barriers. Recent evidence indicated that PFMT is as an effective treatment to reduce UI [14]. Likewise, the advantages of using a mobile application for treatment and users adhered to PFMT long enough for physiological change to occur within the Levator ani, Puborectalis, Pubococcygeus, Iliococcygeus, Coccygeus muscles and supporting fascia and consequently improve QOL [16]. However, a recent review by Hall et al. indicated that pelvic floor strength and contraction duration remain important measurements in pelvic floor assessment, which require internal examination by health professionals or home use internal feedback devices to assess correct activation of these muscles [17]. Physiological outcome measures provide the strongest evidence of a mobile application delivering PFMT for a reduction in UI as effective. Results signify that increased pelvic floor muscle strength-maintained bladder neck support, decreased bladder and urethral pressure maintaining urethral closure and therefore preventing UI, as a direct result of adherence to PFMT via a mobile application [10–13]. Furthermore, another review also reported that both discrepancy and unclear description in the content of PFMT programs make it difficult to assess the efficacy of program in managing UC [17]. Equally as burdensome are the physiological effects of UI and the direct impact on QOL [18]. A review indicated that the financial burden of outlaying costs for a never-ending supply of continence products can escalate individual stress levels, as does the personal shame of living with UI [19].

It is well documented that barriers to health care are detrimental to good outcomes and leaving the offending condition untreated greatly impacts QOL [20]. Pleasingly the results from [10–13] have proven an alternative method to deliver PFMT proving it can overcome barriers and provide the same results as health care led pelvic floor muscle training. The flexibility of a mobile application empowers the patient in their own care, adherence is ensured, and the desired effects of symptom reduction and improved QOL are achieved and all by the touch of a finger.

### Limitation

This review focussed solely on women experiencing UI and there is indication for further research into this area, expanding on the limitations of the earlier studies. Other limitations in general for example, paediatric population, males, non-English studies, or developing countries. This systematic review focussed on randomised controlled trials, the limitation being the small number of trials available. The literature search for this systematic review did not identify any qualitative studies examining in this area of research.

## Conclusion

The effectiveness of mobile applications used to improve UI has been established within the studies included in this review. Adherence is key to PFMT and mobile applications provide flexible training in the home overcoming healthcare barriers. Mobile applications guiding PFMT increase adherence to treatment and reduce UI therefore improving QOL. Future research will contribute to and guide clinical practice.

## Supplementary Information

**Additional file 1.**

**Additional file 2.** Characteristics of Included Studies - Randomized Controlled Trial Form.

**Additional file 3.**

## Data Availability

All data generated or analysed during this study are included in this published article [and its supplementary information file [2]. Datasets are available through the first author upon reasonable request.
