# Supplementary material for: Effectiveness of mobile apps to improve urinary incontinence: a systematic review of randomised controlled trials
Source: BMC Nurs. 2022 Jan 28;21:32. doi: 10.1186/s12912-022-00812-6 (PMC8796429; doi:10.1186/s12912-022-00812-6)
Supplement: Supplementary file 2 — Additional file 2. Characteristics of Included Studies - Randomized Controlled Trial Form. [file 12912_2022_812_MOESM2_ESM.docx]

**Characteristics of Included Studies - Randomized Controlled Trial Form.**

| **Study** | **Country** | **Overall Characteristics** | **Setting/context** | **Participant characteristics** | **Groups** | **Outcomes measured** | **Description of main results** |
| --- | --- | --- | --- | --- | --- | --- | --- |
| Asklund I, Nyström E, Sjöström M, Umefjord G, Stenlund H, Samuelsson E. 2017. | Sweden | To evaluate the effect of a mobile app treatment for stress urinary incontinence (SUI) in women. | Community dwelling women accessing a website. | Women aged 18 years and over with stress urinary incontinence (S.U.I) greater than 1 episode/week for the last 6 months, without urgency and who had access to a smart phone, email, and Swiss literacy. The App group had a mean age of 44.8 years while the control group was 44.7 The mean BMI was 24 in the App group and 24.5 in the control group. Education among the groups was higher in the app group with a mean of 52 and 46 in the control group. Smoking was reported at 2 for the App group and 3 for the control group. Nulliparous was 5 in the App group and 4 in the control group. Those taking regular mediation were higher in the app group at 28 and 24 in the control group. Daily use of incontinence aids was similar with 13 using them in the app group and 14 in the control group. | Women were randomised 1:1 into an RCT with two parallel groups with treatment verse no treatment. The intervention group received an email with instruction for downloading and installing the app. The app group focused on PFMT and exercises were ordered at 6 basic and 6 advanced levels. PFME included different combinations and repetitions including basic, quick and pre-coughing contractions three times per day. The app provided them the option to set an alarm to remind them. Data would be saved in a statistic table within the app. The goal was for the women to complete these in 3 months. The control group was a postponed treatment group. The control group received the app after the initial 3 month follow up of the app group. Sample size was calculated form a previous internet study size aiming for a sample size of initially 60 participants in each group with an expected 33% drop-out rate. | The primary outcome measures were symptom severity (ICIQ-UI SF) and condition-specific quality of life (ICIQ-LUTqol). The secondary outcomes included the Patients Global Impression of Improvement (PGI-I) a self-rated validated questionnaire asking about changed experience after treatment. The incontinence episodes frequency (IEF) was also measured. Incontinence aids were estimated for both groups over the previous 4 weeks, before baseline and before follow-up | One hundred and twenty-three women were included in the study with moderate/severe LUTS (97.5%, 120/123), mean ICIQ-UI SF score 11.1 and mean ICIQ-LUTSqol score was 34.4 at baseline. At follow-up, the app group reported improvements in symptom severity and condition specific quality of life. In the app group 98.4% (60/61) performed PFMT at follow-up and 41.0% performed it daily. Overall, the app treatment was effective for women with S.U.I, yielding clinically relevant improvements. |
| Araujo CC, Marques AA, Juliato CRT. 2019. | Brazil | To evaluate the use of a mobile device application (app) for the treatment of urinary incontinence through adherence to home pelvic floor muscle training (PFMT) and its impact on urinary symptoms. | Physiotherapist clinic of the University of Campinas. | 33 women with self-reported urinary incontinence symptoms were included in the randomized 1:1 single-blind parallel study. 17 women were allocated to the APP group and 16 women allocated to the control group. The mean age of the APP group was 47.2 and the mean age of the control group was 53.3. Within the app group 13 identified as Caucasian and 4 as other. Within the control group 14 identified as Caucasian and 2 as other. The mean BMI in the APP group was 27.9 and 28.5 in the control group. There were 3 smokers in the APP group and 1 in the control group. A sedentary lifestyle was documented in 10 within the APP group and 9 in the control group. Mean pregnancy in the APP group was 3 and 3.7 in the control group. | The APP group of 17 women received the pelvic floor muscle exercise app. The 16 women in the control group received printed instructions. | The primary outcome was to measure adherence to pelvic floor muscle exercise with one compete sequence including 8x hold/relaxation and contraction. The secondary outcome measured were changes in vaginal symptoms, quality of life, urinary and stress urinary symptoms obtained through questionnaires. Pelvic floor muscle examination of power, endurance, number of repetitions and fast contractions. Cure rates were also a secondary outcome. | Baseline evaluation by PERFECT scheme it was observed that women from the control group had lower initial P values than the APP group. Adherence (number of repetitions of entire protocol) was higher in the APP group at 1 & 2 months after PFMT. Adherence decreased, especially in the control group at 1,2 & 3 months. The women self-reported adherence rate. The APP group showed better results during treatment. Secondary outcome results little difference in both groups, however the PERFECT scheme found improvement was superior in the APP group. In total 91% of women in both groups reported symptom improvement after PFMT, with only 2 women from the app reporting unchanged symptoms and no-one in the control group. |
| Wang X, Xu X, Luo J, Chen Z, Feng S. 2020. | China | To determine the effectiveness of app-based audio guidance pelvic ﬂoor muscle training on the treatment of stress urinary incontinence in primiparas. | The study was conducted in the obstetric clinic of a tertiary maternity hospital in Hangzhou China. | 108 eligible primiparas were recruited. Nulliparous women who were continent prior to and with a singleton pregnancy and cephalic presentation at 30-32 gestational age, and 20-34 years old with an episode of stress urinary incontinence once per month and within 3 months. Baseline demographics, characteristics and obstetrical variables were similar between the groups. | 54 women were allocated to the intervention group and received audio guidance training. They were guided to download and install the app. The app contained a home training module, a systematic audio guidance pelvic floor muscle training program. participants were guided by audio and music synchronized throughout the training. They received 45 min pelvic floor rehab education and one on one PFMT practice guidance.54 women were allocated to the control group and received conventional training. They received 45 min pelvic floor rehab education and one on one PFMT practice guidance. | Primary outcomes measured were symptom severity score and adherence to pelvic floor muscle training, which was assessed at 6 weeks, 3 months, and 6 months postpartum. Secondary outcomes were pelvic floor muscle strength assessed by surface electromyography and vaginal palpation, bladder neck mobility by perineal ultrasound and sexual function with the Female sexual function index (FSFI) | Participants showed signification improvement in symptom severity across the study, which was most significant at 6 weeks postpartum. There was no significant difference in symptom severity between groups. The interaction effect between intervention and time on adherence was significant. Compared to the control group, greater self-efficacy was shown in the audio group at 6 weeks, 3 months, and 6 months postpartum. Participants in the audio group indicated less bladder neck descent at 6 weeks postpartum, better pelvic floor muscle strength and sexual function at 6 months postpartum. |
| Hoffman V, Söderström L, Samuelsson E, Söderström L. 2017. | Sweden | To determine the effectiveness of app-based audio guidance pelvic ﬂoor muscle training on the  treatment of stress urinary incontinence in primiparas. | Community dwelling women who participated in the original trial who were part of the app group were contacted via email with a link to a web survey. | The average age of the 46 participants who responded at 2 years to follow-up was 44.2 years. BMI 23.7, 37 attended university, 1 was a smoker. | The intervention group who used the app for the RCT 2 years previous was the only group included in this follow up. 46 participants completed the follow-up.The control group was excluded from this 2 year follow-up as they did receive the app 3 months after the intervention group, however they were not followed for the next 3 months after this and consequently no data was collected for their post treatment outcomes using the app. | Primary outcomes measured were the ICIQ-UI SF and the ICIQ-LUTSqol. Secondary outcomes measured included answers to the PGI-I and the use of incontinence products. | The difference in score from baseline to the 2-year follow-up was the primary outcome. Of the 62 women, 61 and 46 respectively participated in three month and two-year follow-ups. Baseline data did not differ between responders and non-responders at follow-up. The mean decreases in ICIQ-SF & ICIQ-LUTSqol scores were 3.1 and 4.0respectively. Of the 46 women, 4 rated themselves as very much better, 9 as much better, and 16 as a little better. The use of incontinence products decreased significantly, and the proportion of women felt they could contract their pelvic muscles correctly increased from 14/46 at baseline to 31/46 at follow-up. Self-management of SUI with a mobile app had significantly relevant long-term effects and may serve as first line treatment. |
