# Supplementary material for: Effectiveness of mobile apps to improve urinary incontinence: a systematic review of randomised controlled trials
Source: BMC Nurs. 2022 Jan 28;21:32. doi: 10.1186/s12912-022-00812-6 (PMC8796429; doi:10.1186/s12912-022-00812-6)
Supplement: Supplementary file 3 — Additional file 3. [file 12912_2022_812_MOESM3_ESM.docx]

| Citation | **Intervention** | **Outcomes Measured** | Features included |
| --- | --- | --- | --- |
| Asklund et al  (2016) | The mobile app Tät with a treatment program focused on pelvic ﬂoor muscle training (PFMT), and information about stress urinary incontinence and lifestyle factors.  The app group received an e-mail with  instructions for downloading and installing the app from the  App Store or Google Play and a code to open the app.  3 months follow up | Symptom severity  Condition-speciﬁc quality of life | 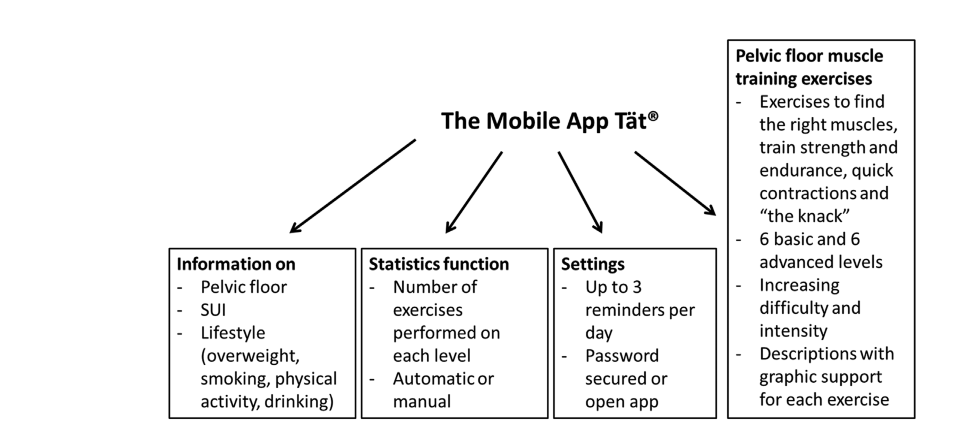 |
| Araujo et al (2019) | The mobile app  1, 2 and 3 months follow up | Adherence  Quality of life, urinary and stress urinary symptoms | 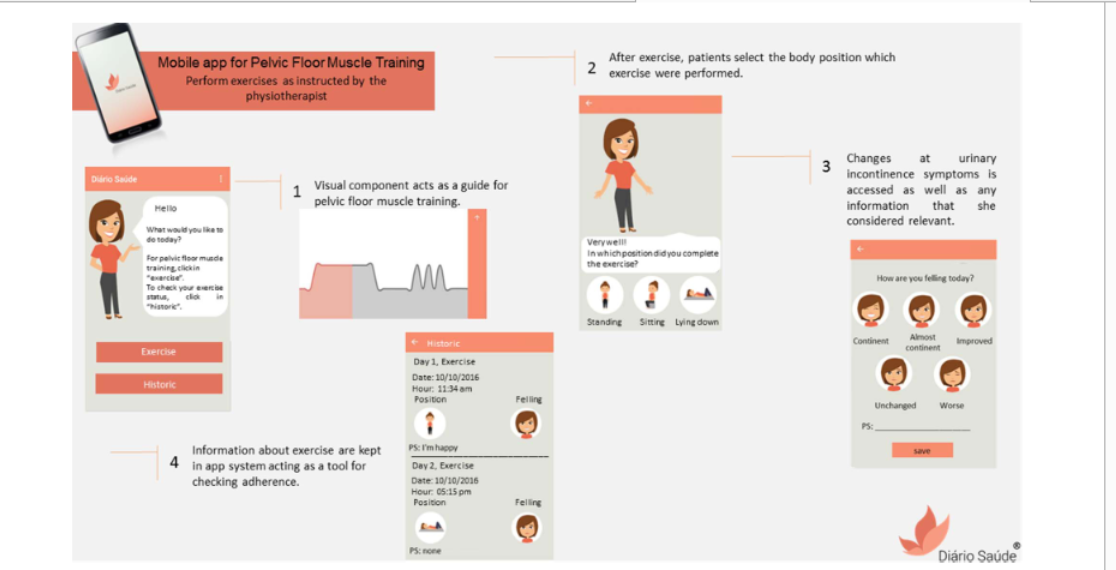 |
| Hoffman et al  (2017) | The mobile app Tät  3 months follow up  Two years follow up | Symptom severity  Condition-speciﬁc quality of life | Not reported |
| Wang et al (2020) | Mobile phone app  6 weeks follow up  3 months follow up | Severity of stress urinary incontinence and adherence to pelvic ﬂoor muscle training | Not reported |
